# 145. Tracheal Aspirate Order Indications and Associated Culture Results in Mechanically Ventilated Children

**DOI:** 10.1093/ofid/ofad500.218

**Published:** 2023-11-27

**Authors:** Monika Jelic, Matthew J Weber, Meghan C Birkholz, Elaine Dowell, Stacey Hamilton, Sarah A Jung, Christopher D Baker, Sarah K Parker, Samuel R Dominguez

**Affiliations:** University of Colorado School of Medicine, Aurora, CO; University of Colorado School of Medicine, Aurora, CO; Children's Hospital Colorado, Clifton, Virginia; Children's Hospital Colorado, Clifton, Virginia; Children's Hospital Colorado, Clifton, Virginia; Children's Hospital Colorado, Clifton, Virginia; University of Colorado School of Medicine, Aurora, CO; University of Colorado/Children's Hospital Colorado, Aurora, CO; University of Colorado School of Medicine, Aurora, CO

## Abstract

**Background:**

Tracheal aspirates (TAs) are collected on mechanically ventilated patients to aid in diagnosis of tracheitis or ventilator associated pneumonia. Accuracy of TA cultures to diagnose lower respiratory tract infections is low, limited by normal respiratory flora contamination, making interpretation difficult. To aid diagnostic stewardship efforts, we analyzed TA order indications to better understand why TA cultures were ordered and which indications were associated with a clinically significant culture result.

**Methods:**

Order indications for TA specimens were implemented in Sept. 2021 at Children’s Hospital Colorado. At least one indication was required at the time of order placement, Table 1. We extracted all TA orders from Sept. 2021 to Aug. 2022, as well as demographics, collection department, and Gram stain/culture results.

**Results:**

1,439 tracheal aspirate specimens were ordered for 496 unique patients. 34% were collected in the Emergency Department (ED), 22% in the Neonatal Intensive Care Unit (NICU), and 20% in the Pediatric Intensive Care Unit (PICU; Table 1). The two most common order indications were increase in secretions (37%) and increase in ventilator support (36%). 296 (22%) cultures grew 1 or 2 predominant organisms, with the most common indication being increased secretions; 57% of these cultures grew respiratory pathogens. Overall, 42% of cultures grew normal respiratory flora and 12% had no growth. For cultures with no growth and those with no organisms on Gram stain, the most common indication was increase in ventilator support. For all order indications, 50% or more grew normal respiratory flora with abnormal chest imaging and fever having the most cultures with 1 or 2 predominant organisms.

**Figure:**
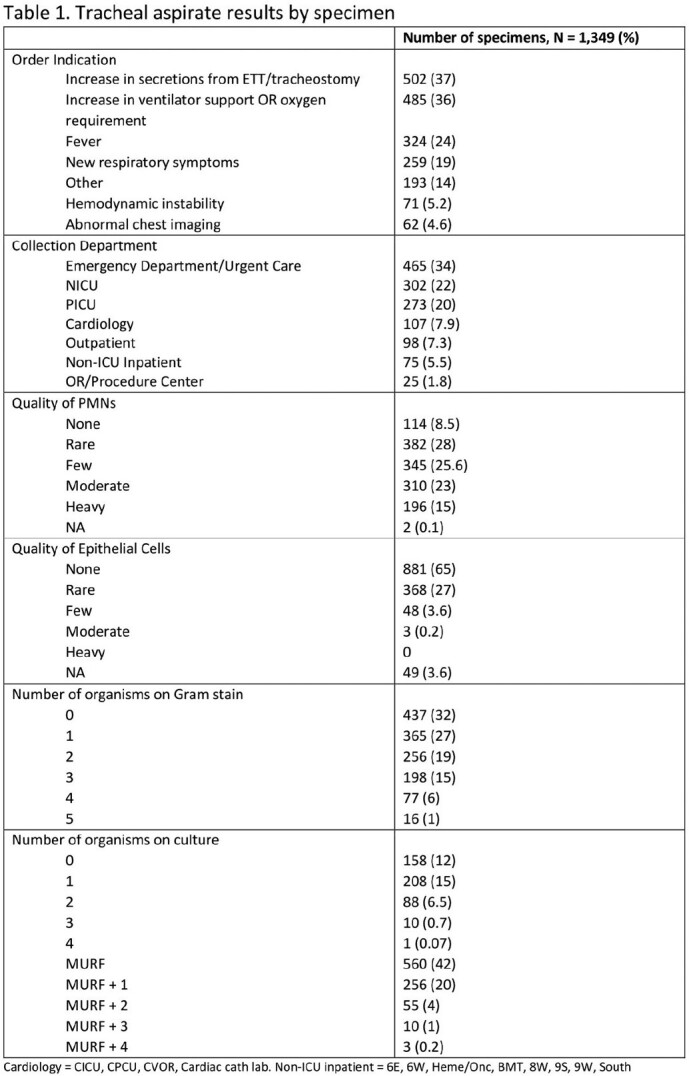

**Figure:**
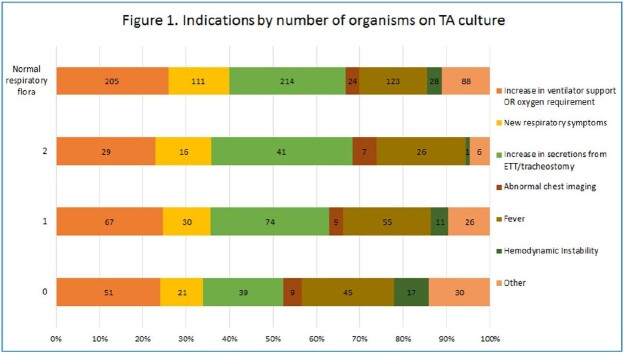

**Conclusion:**

The majority of TAs are collected in the ED, NICU or PICU. The most common order indications were increase in secretions or increase in ventilator support. The most common order indication that was noted in actionable cultures (1 or 2 predominant organisms) was increased secretions, though order indication was not predictive. These results have informed potential Gram stain rejection criteria to improve diagnostic yield for clinically relevant pathogen detection.

**Disclosures:**

**Sarah A. Jung, PhD**, Abbott Molecular Inc: Research support|DiaSorin Molecular: Research|Karius: Advisor/Consultant|Karius: Industry talks at two conferences **Samuel R. Dominguez, MD, PhD**, Biofire Diagnostics: Advisor/Consultant|Biofire Diagnostics: Grant/Research Support|Cobio Diagnostics: Board Member|Karius: Advisor/Consultant|Pfizer: Grant/Research Support

